# Evaluation of Beta-Catenin Subcellular Localization and Water Channel Protein AQP1 Expression as Predictive Markers of Chemo-Resistance in Ovarian High-Grade Serous Carcinoma: Comparative Study between Preoperative Peritoneal Biopsies and Surgical Samples

**DOI:** 10.3390/diagnostics11030452

**Published:** 2021-03-05

**Authors:** Giuseppe Angelico, Antonio Ieni, Rosario Caltabiano, Angela Santoro, Frediano Inzani, Saveria Spadola, Giovanni Tuccari, Antonio Macrì, Gian Franco Zannoni

**Affiliations:** 1 Fondazione Policlinico Universitario A. Gemelli IRCCS, Dipartimento Scienze della Salute della Donna, del Bambino e di Sanità Pubblica, Unità di Gineco-patologia e Patologia Mammaria, 00168 Roma, Italy; giuangel86@hotmail.it (G.A.); angela.santoro@poiclinicogemelli.it (A.S.); frediano.inzani@policlinicogemelli.it (F.I.); saveriaspadola@hotmail.it (S.S.); 2 Department of Human Pathology in Adult and Developmental Age “Gaetano Barresi”, Section of Pathology, University of Messina, 98122 Messina, Italy; aieni@unime.it (A.I.); tuccari@unime.it (G.T.); 3 Department Gian Filippo Ingrassia, Section of Anatomic Pathology, University of Catania, 95123 Catania, Italy; rosario.caltabiano@unict.it; 4 Department of Human Pathology in Adult and Developmental Age “Gaetano Barresi”, Section of Surgery and Peritoneal Surface Malignancy and Soft Tissue Sarcoma Program, University of Messina, 98122 Messina, Italy; amacri@unime.it; 5 Istituto di Anatomia Patologica, Università Cattolica del Sacro Cuore, 00168 Rome, Italy

**Keywords:** ovarian cancer, serous carcinoma, chemo-resistance, pathologic response, platinum-based chemotherapy, aquaporin 1, β-catenin, wingless-related integration site (wnt)/β-catenin pathway

## Abstract

**Background.** Mutations of the β-catenin gene (CTNNB1), leading to aberrant immunohistochemical expression of β-catenin, represent a key mechanism of WNT/β-catenin pathway alteration in ovarian cancer. Aquaporin 1 (AQP1), as component of transmembrane-water-channel family proteins, has been documented in different human tumors and, recently, also in ovarian carcinoma. Only few studies have investigated the pathogenetic and prognostic role of β-catenin and AQP1 in ovarian carcinoma. Methods. We evaluated the expression of β-catenin and AQP1 in the preoperative peritoneal biopsies of 32 patients with peritoneal carcinosis, in which a histological diagnosis of high grade serous ovarian carcinoma was made. Furthermore, we have investigated their potential association with chemotherapeutic response evaluated at the omental site, as well as with clinico-pathological parameters. Results. Sixteen cases showed an aberrant membranous and cytoplasmic β-catenin staining pattern. The remaining 16 cases showed a preserved β-catenin expression localized only in cell membranes; 20 cases showed positive membranous staining (AQP1+), while 12 cases were considered negative (AQP1–). In the AQP+ group, we detected a significant association of AQP1 expression with poor chemotherapy response in omental tissues complete response score (CRS) 1-2, while a CRS 3 was never observed in all positive cases. Conclusions. Our findings suggest that β-catenin and AQP1 are expressed in a sub-group of ovarian tumors and play important roles in carcinogenesis. Patients affected by high grade serous carcinoma could be categorized in two different predictive groups: as AQP+ and AQP–. AQP+ cases may represent a subset of poor responders who could be considered more eligible for cytoreductive surgery rather than for neoadjuvant chemotherapy.

## 1. Introduction

Epithelial ovarian carcinomas (OC) represent the most lethal gynaecological malignancy, being the fifth cause of female related cancer death [1]; its incidence and mortality are constantly increasing, mainly because the majority of women are diagnosed in advanced stage [1]. To date, primary debulking surgery and neoadjuvant chemotherapy (NACT) followed by surgery (interval debulking surgery, IDS) represent the mostly accepted treatment options for OC [2]. Moreover, NACT and IDS have been proposed ever more for OC patients to increase the radicality of surgery and to reduce morbidity and mortality, taking into consideration the favorable results obtained in two randomized controlled phase III trials [3,4]. However, it is well known the histopathological assessment of NACT response in OC represents the most important prognostic tool to establish the rate of complete citoreductive surgery and to predict patient outcome [5,6,7].

Currently, the complete response score (CRS) system proposed by Böhm et al. is considered the most reliable histopathological grading system for assessing NACT response in OC [8]. Specifically, it consists of a three-tier CRS based on the evaluation of omental residual disease, which shows a good correlation with progression-free survival and overall survival: Score 1: No or minimal tumor response; Score 2: Partial tumor response; Score 3: Complete or near-complete response [8]. Moreover, the International Collaboration on Cancer Reporting recommended the use of the Böhm’s CRS system for grading the NACT response in OC, also as the consequence of confirms and validations by other authors [7,9,10,11,12]. Despite the considerable advances in the pre-operative diagnosis and treatment strategies, the majority of OC still recur and develop chemoresistance with poor 5-year survival [1,13]. Therefore, new prognostic biomarkers are needed to predict the biologic behavior and therapeutic response, improving the clinical outcomes of OC patients. In this field, some previous studies highlighted the potential role in carcinogenesis, tumor progression and metastasis development of different cancers by Aquaporin 1 (AQP1), a small trans-membrane water channel protein [14,15,16,17,18,19].

Several studies demonstrated the AQP1 role in cell proliferation, adhesion, and motility, as well as in the modulation of serous fluid volumes. Moreover, increased immunohistochemical expression of AQP1 in bioptic and surgical have been reported to predict improved overall survival (OS) in patients affected by mesothelioma, HER2-positive early breast cancer, colorectal cancer, and biliary tract carcinoma. On the other hand, increased levels of AQP1 have been associated with a poorer prognosis in brain tumors, prostate adenocarcinoma, lung adenocarcinoma, and carcinomas of the gastrointestinal tract [14,15,16,17,18,19]. Only few studies have investigated the prognostic role of AQP1 expression in EOC [20,21,22,23,24,25,26]. In detail, when ovarian carcinomas were divided by histological types, low AQP1 expression correlated with poorer prognosis in clear cell variant, while high AQP1 content has been related to poorer prognosis in mucinous and endometrioid carcinomas [26]. Moreover, recent studies suggest that the Wingless-related integration site(Wnt)/β-catenin pathway regulates many key aspects of cancer development, including cell proliferation, cancer stem cells (CSCs) survival; metastasis and chemoresistance [27,28,29,30]. Mutations of the β-catenin gene (CTNNB1), leading to aberrant immunohistochemical expression of β-catenin, have been reported in all subtypes of OC and represent a key mechanism of WNT/β-catenin pathway alteration [27,28,29,30]. Consequently, the aim of the present study is to analyze the β-catenin and AQP1 expression in serous high-grade advanced OC and their potential relationship with response to chemotherapy, in order to verify whether these antibodies may be considered as additional useful prognostic biomarkers in OC patients. 

## 2. Materials and Methods

The study complied with the Ethical Principles for Medical Research Involving Human Subjects according to the World Medical Association Declaration of Helsinki; the non-interventional, retrospective nature of our study did not require informed consent; however, a written informed consent was obtained from patients before surgical procedures. Patients’ medical records and pathology reports were utilized to obtain clinical data. Patients’ initials or other personal identifiers were not shown in images. All samples were anonymized and no further ethical approval was necessary to perform the retrospective study.

### 2.1. Patient Selection and Clinical Data

A cohort of 32 patients presenting with peritoneal carcinosis documented by diagnostic peritoneal biopsies which confirmed the histological diagnosis of high grade serous ovarian carcinoma (HGSC) was included in the study. All patients met the following additional inclusion criteria: International Federation of Gynecology and Obstetrics (FIGO) stage IIIC/IV, platinum-based NACT, and complete clinical response after neoadjuvant chemotherapy: score 0 according to the surgical scoring system for the IDS residual disease (0, no residual disease; 1, ≤1 cm residual disease; 2, >1 cm residual disease; 3, Unknown). All selected patients, on the basis of clinical, serologic, instrumental data, and/ or surgical exploration were considered as non-eligible for primary debulking surgery. IDS was performed either by laparotomy or minimal invasive surgery according to pre-operative evaluation, preference and experience of surgeons. After surgical procedures, all patients were routinely evaluated with clinical visits and CT-scan examination after three cycles of NACT and the IDS was proposed after the third cycle, if there was any evidence of progressive disease. After concluded initial treatment, the follow-up was scheduled for all patients, every 3–4 months for 2–3 years and successively, every 6 months for the next 3 years.

### 2.2. Pathology Evaluation

The histological CRS following IDS was determined in the omental sites according to the three-tiered CRS proposed by Böhm et al. [7,8]. All the omental and ovarian formalin-fixed paraffin embedded tissue blocks were sectioned at 4–5 μm intervals, stained with haematoxylin and eosin (H&E) and reviewed by a team of experienced pathologists (GFZ, AS, GT and GA), who were blind of clinical data and each other results to assign the CRS of 1–3 for the omental samples. Fleiss-Cohen weighted k statistics were used to assess the concordance rate of CRS in high grade OC. *k* values ranging between 0 and 0.2, 0.21 and 0.4, 0.41 and 0.6, 0.61 and 0.8, 0.81 and 1 were considered as no agreement, fair agreement, moderate agreement, substantial agreement, and perfect agreement, respectively.

CRS was evaluated as follows: Score 1: absent or minimal tumor response (viable tumor with minimal regression-associated fibro-inflammatory reaction, limited to isolated foci); Score 2: Partial tumor response (multifocal or diffuse regression associated fibro-inflammatory reaction, with easily identifiable residual tumor); Score 3: Complete or near-complete response (prevalent regression, with few irregularly scattered residual tumor cells or cell groups, all measuring <2 mm, or no residual tumor identified) [7,8]. If there was no agreement between observers, slides were jointly discussed by using a double-headed microscope, until agreement was reached.

### 2.3. Immunohistochemistry

β-catenin and AQP1 immunohistochemistry were evaluated in preoperative peritoneal diagnostic biopsies from all patients before they received chemotherapy. In this way, we ensured that our immunohistochemical results were not altered by drug-changes in tissue samples. Four-five μm thick sections were cut, mounted on xylane-coated slides (Dako, Glostrup, Denmark), stained with hematoxylin and eosin (H&E) and examined using a Zeiss Axioplan light microscope (Carl Zeiss, Oberkochen, Germany) for a preliminary morphological evaluation, avoiding the presence of structural alterations. Moreover, on parallel sections, AQP1 (B-11, Santa Cruz Biotechnology, Santa Cruz, CA, USA; dilution 1:100) and β-catenin (clone β-Catenin-1, DAKO, dilution 1:100) antibodies were applied using a Ventana Benchmark immunostainer (Ventana Medical Systems, Inc., Oro Valley, AZ, USA); m-IgGκ BP-HRP (mouse IgGκ binding protein-HRP) were utilized as secondary detection reagents for β-catenin and AQP1 antibodies. The reaction was then visualised with 3-3’ diaminobenzidine tetrahydrochloride, and the slides were counterstained with Mayer’s haemalum.

Three patterns of β-catenin expression were evaluated in cancer cells: cytoplasmic: normal β-catenin localization in cell cytoplasm; membranous, if β-catenin was localized in the cell membrane; nuclear: β-catenin expression in the nucleus. β-catenin expression was considered aberrant when cytoplasmic and/or nuclear staining was recorded in ≥10% of tumour cells, as elsewhere reported [30].

**Figure 1:**
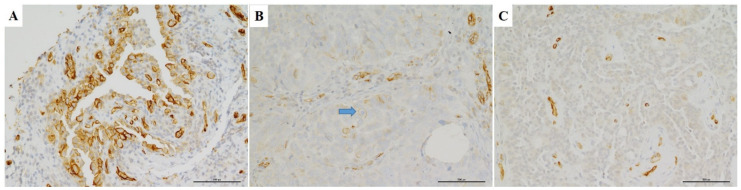
Different Immunohistochemical expression patterns of AQP1 in diagnostic biopsies of high grade serous ovarian carcinomas. (**A**) Diffuse positivity for AQP1 showing linear and circumferential membranous staining is depicted. (**B**) Another serous carcinoma case showing focal staining for AQP1. Arrow indicates a neoplastic cell with linear and circumferential membranous staining. (**C**) Negative staining for AQP1 is depicted. Arrow indicates vascular endothelial cells which served as positive internal control. (A-B-C: IHC, LSAB-HRP, 20×).

For AQP1, only membrane labelling was considered specific since it has been previously demonstrated as the most reliable staining pattern in different solid tumors [17,18,19,20,21]; this pattern of labelling was confirmed from 10 high-power (400×) fields (Figure 1A). Positive and negative controls for AQP1 were included to test the specificity of the immunoreaction. Endothelial cells and mesothelial cells were considered as positive internal controls (Figure 1B); for negative controls, the primary antiserum was omitted and replaced by non-immune serum or phosphate buffered saline solution (pH 7.6). Representative photomicrographs were then captured using a digital camera (AxioCam MRc5, Carl Zeiss, Thornwood, NY, USA). Therefore, as elsewhere suggested [17,18], the percentage of immunostained cells was assessed by semi-quantitative optical analysis according to a four-tiered system (0 = negative; > 1% to 24% positive cells = focal staining; ≥25 to <49% positive cells = not uniform staining; ≥50% positive cells = diffuse staining). Cases showing a value more than >1%, as the median of immunoreactive neoplastic cells, were considered positive for AQP1 expression.

### 2.4. Statistical Analysis

To assess the predictive value of AQP1 for omental residual disease, Fisher exact test was performed using the SPPS Statistics 23 software (SPSS Inc, New York, NY, USA). Chi-square test was used to analyze associations between high and low AQP-1 expression and clinico-pathological parameters including age, stage, CRS, and outcome. A *p* value less than 0.05 was regarded as statistically significant. Sample size was determined in order to achieve a power of 0.80, an alpha of 0.05 and the hazard ratio of 2 between the two groups. Cancer-specific survival analysis was performed using the Kaplan–Meier method, and Mantel–Cox log-rank test was used for comparison of the survival curves.

## 3. Results

### 3.1. Patient Baseline Characteristics

Thirty-two women (mean age 62 years, age range 42–86 years) with advanced stage IIIC-IV ovarian high-grade serous carcinoma treated with neoadjuvant chemotherapy and interval debulking surgery were identified and included in the study. All patients were considered score 0 according to the surgical scoring system for the IDS residual disease.

Moreover, 27 patients had stage IIIC disease, and 5 had stage IV disease. In our study cohort, 10, 17, and 5 patients had omental CRS of 1, 2, and 3, respectively. The k value for the CRS in high grade OC among different observers was 0.87 (almost perfect agreement). All clinico-pathological and immunohistochemical data are analytically summarized in Table 1.

### 3.2. Immunohistochemistry

Immunohistochemical expression of AQP1 was demonstrated by the linear (partial) and/or circumferential, complete membranous staining (Figure 1A). Taking into consideration a cut-off of ≥ 1 % positive tumor cells, 20 (62.5%) cases showed positive AQP1 staining (AQP1+), while 12 (37.5%) cases were considered negative (AQP1–) (Figure 1C). In detail, positive cases were immunohistochemically scored as follows: diffuse (6 cases), not uniform (4) and focal (10).

**Figure 2:**
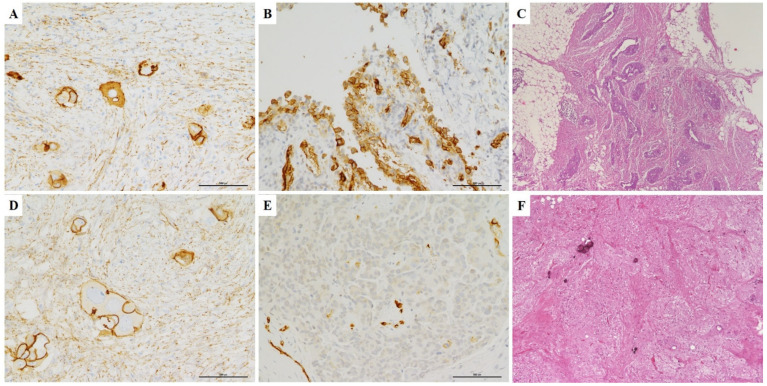
Omental pathological response according to β-catenin and AQP1 IHC. (**A**,**B**) Diagnostic biopsy of a case of high grade serous ovarian carcinoma demonstrating aberrant membranous and cytoplasmic staining for β-catenin (**A**) and diffuse positivity for AQP1 (**B**). (**C**) After NACT and IDS, this case showed an omental response score of 1: mainly viable tumor with no or minimal regression-associated fibro-inflammatory changes. (**D**–**E**) Another serous ovarian carcinoma case showing normal membranous staining for β-catenin (D) and negative staining for AQP1 (**E**). (**F**) After NACT and IDS, this case showed an omental response score of 3: extensive fibro-inflammatory changes with no residual tumor identified (A-B-D-E: IHC, LSAB-HRP, 20×); (C-F: IHC, H&E, 20×).

Regarding β-catenin immune-expression, 16 cases showed an aberrant membranous and cytoplasmic staining pattern. The remaining 16 cases showed a preserved β-catenin expression localized only in cell membranes (Figure 2A,D). Moreover, nuclear staining for β-catenin was never observed in our series.

### 3.3. Omental Chemotherapy Response

In our study cohort, 10, 17, and 5 patients had omental CRS of 1, 2, and 3, respectively. In the AQP1+ group, the statistical analysis (Fisher exact test, SPSS statistical software, New York, NY, USA) showed a significant association of AQP1 expression with poor chemotherapy response in omental tissues CRS1-2 (*p* = 0.0039). In fact, all positive cases showed an omental response score of 1 and 2 (Figure 2B,C), while a complete response score (CRS3) was never observed (Table 2). By contrast, in the AQP1–group, 5 cases showed a complete pathological omental response (Figure 2E,F), while 7 cases were considered as poor responders (CRS1-2). On the other hand, no significant associations emerged between β-catenin membranous or cytoplasmic expression and chemotherapy response in omental tissues (*p* = 0.3326).

### 3.4. AQP1 and β-Catenin Immuno-Expression Relationships

In our series, the statistical analysis (Fisher exact test) showed a significant association between aberrant β-catenin localization and AQP1 immunohistochemistry (*p* = 0.0091). In detail, 14/16 OC patients with aberrant membranous and cytoplasmic β-catenin staining pattern showed also positive immunostaining for AQP1 (Table 1). By contrast, 10/16 OC patients with retained membranous β-catenin staining showed negative staining for AQP1.

### 3.5. Clinico-Pathological Characteristics

**Figure 3:**
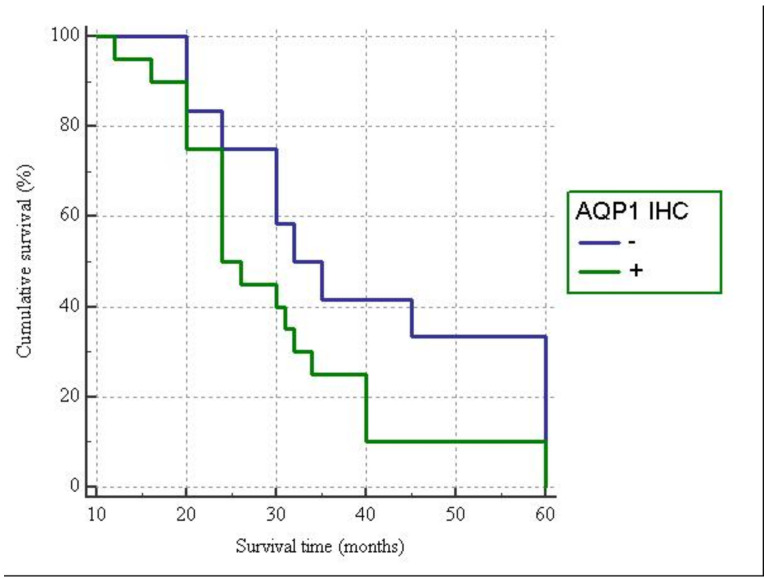
Survival curves of all cases of ovarian high-grade serous carcinomas in relation to immunohistochemical expression of AQP1.

The follow-up of patients ranged from 12 to 60 months (mean follow-up 33.65 months). During the follow-up observation period, nine patients died of the disease, while the remaining twenty-three patients were still alive at the end of the observation period. No significant relationship emerged between β-catenin or AQP1 expression and other clinico-pathological variables; only a statistical trend has been observed for the patient’s age. Among younger patients (<50 aa) we more frequently noted loss of AQP1 expression. Finally, The Kaplan–Meier survival curves, documenting patient survival times stratified according to the AQP1 immunostaining showed a moderate, but not statistically significant, difference in survival rates between positive and negative cases. In detail, starting from the initial pathological diagnosis, the AQP1– and AQP1+ groups showed a median survival time of 32 and 24 months, respectively (*p* = 0.1012) (Figure 3).

## 4. Discussion

Accumulating evidence supports the key role of the Wnt/β-catenin pathway in OC development, in particular in endometrioid and mucinous histotypes [27,29,30]. Moreover, altered expression of beta-catenin or alterations in subcellular location (nuclear/cytoplasmatic vs. membrane) has also been demonstrated in HGSC, where mutations in Wnt-related genes are relatively uncommon [27,29,30]. Therefore, deregulation of Wnt signaling and the consequent delocalization of beta-catenin may contribute to HGSC progression. However, data regarding its potential role in carcinogenesis, in predicting chemoresponse or in its prognostic relevance are still limited. In the present study, we documented an aberrant beta-catenin staining pattern in half of our cases. In detail, a membranous and cytoplasmic aberrant staining pattern was observed in 16/32 OC patients while the remaining 16 cases showed a maintained membranous staining. Moreover, nuclear staining was never observed. These results are in line with the few previous observations of beta-catenin staining in OC [30].

In our study, we have not performed molecular studies to detect mutations of the relevant genes involved in canonical WNT pathway, neither we have found nuclear accumulation of β-catenin by immunohistochemistry, but all the cases showed high level of membranous or mixed membranous and cytoplasmic protein expression in at least 60% of tumor cells. This finding suggested two important concepts: (1) β-catenin overexpression can be heterogeneous; (2) The molecular mechanisms and down-stream effects of non-nuclear β-catenin overexpression may be different from those with nuclear protein accumulation and may be independent of the Wnt signaling pathway [27].

Moreover, several studies demonstrated that non-nuclear type β-catenin overexpression appeared to have pathologic and prognostic significance in different solid tumors including hepatocellular carcinoma and oropharingeal cancer [28,31,32].

However, we failed to observe significant associations with aberrant beta-catenin staining and clinicopathological features such as patient’s outcome and chemotherapy response. This may be explained by the small sample size and by the possible interactions of other Wnt pathway molecules in the HGSOC pathogenesis and platinum-resistance mechanism [27]. Therefore, we retain that further investigations are required in order to elucidate the molecular mechanisms regulating non-nuclear subcellular localization of β-catenin and to demonstrate its prognostic relevance.

Interestingly, we observed a significant association between aberrant β-catenin expression and AQP1 immunohistochemistry (*p*= 0.0091). In fact, the aberrant membranous and cytoplasmic β-catenin staining pattern observed in the AQP1+ group, may suggest an interaction of these two proteins in HGSC carcinogenesis. This positive correlation between AQP1 and β-catenin expression has already been documented in other tumor types and, in our opinion needs to be furtherly investigated in OC [33,34].

AQP1 analysis has been investigated in several neoplastic tissues, in which a significant association between its expression, tumor phenotype and survival outcomes has been documented [14,15,16]. In particular, the high AQP1 expression has been associated with poor prognosis in numerous cancers, including ovarian carcinoma, lung cancer, prostate adenocarcinoma, brain tumors and breast cancer [14,15,16,19]. By contrast, AQP1 high expression in mesotheliomas is associated with improved survival rates, as elsewhere by us reported [17,18].

Recently, in a gynaecological context, some Authors have immunohistochemically evaluated the expression of AQP1, 3, 5, and 9 in a total of 300 ovarian carcinomas using tissue microarrays, by demonstrating that AQPs can be considered useful prognostic markers in ovarian carcinoma [26]. However, the correlation with prognosis depends on the histological type of ovarian carcinoma; specifically, high AQP5 expression is related to poorer prognosis in serous carcinoma, while low AQP1 expression was evident in clear cell carcinomas with poorer prognosis [26]. Moreover, high AQP1 expression is associated with poorer prognosis in mucinous and endometrioid carcinomas [26].

Although controversial results are reported concerning AQP1 expression and tumor progression or metastasis development, only few data are available in the literature regarding the association between AQP1 and response to chemotherapy [23]. Recently, in patients with stage II–III colorectal cancer treated 5-FU (fluorouracil)-based adjuvant chemotherapy, positive AQP1 expression was associated with an increased disease free survival (DFS) rate compared with that of AQP1-negative ones [35]; therefore, it has been suggested that AQP1 may be a candidate biomarker predictive of response to 5-fluorouracil-based adjuvant chemotherapy [33]. Furthermore, in prostatic adenocarcinoma cell lines, AQP1 was suppressed by ginsenoside Rg3, together with cell migration [36]. A down-regulation of AQP1 has been reported in lung cancer cell lines treated by combination therapy of celecoxib and afatinib [37]. Moreover, different subtypes of AQPs play different roles in ovarian cancer cell in vitro, suggesting thus AQPs might be associated with chemotherapy sensitivity [30]. In detail, the cisplatin effects were different between since the expression of AQP1 mRNA decreased significantly, while expression of AQP3 and AQP8 increased [23].

In the present study, we investigated the immunohistochemical expression of AQP1 in pre-operative peritoneal samples obtained from advanced stage serous OC. We have shown that a sub-group of these OC exhibited an evident immunohistochemical AQP1 expression in comparison to a negative one. Although no relationship between clinico-pathological parameters and AQP1 has been encountered in our cohort, we have thought to be of interest to verify if AQP1 expression is able to predict the chemotherapy response following NACT and IDS. In detail, evaluating the omental tissues chemotherapy response, a significant association was observed between AQP1 expression and poor chemotherapy response CRS1-2; in addition, a complete response score (CRS3) was never noted in AQP1+ patients. Consequently, it may be hypothesized that AQP1 could represent a useful predictive biomarker of tissue response to platinum-based chemotherapy in patients affected by high grade serous OC.

Finally, accordingly to previous observations regarding the relationship between AQ1 and patient outcome in carcinomas of different sites, such as ovary, lung, prostate, brain, and breast [14,15,16,19], we have documented a sensible trend for better survival in patients with negative AQP1 immunoexpression.

## 5. Conclusions

In our study, the aberrant β-catenin staining pattern observed in the AQP1+ group supports a possible interaction of these two proteins in ovarian carcinogenesis. Similarly, recent in vitro studies demonstrated how β-catenin is involved in AQP1 mediated cell migration [34,38]. Moreover, AQP1 has been demonstrated to act as a scaffold for plasma-membrane associated multiprotein-complex necessary for cell integrity, adhesion, and motility. However, the exact functional roles and the prognostic value of their combination have not been fully elucidated up to now.

Our data may stimulate future research in expanding the comprehension of platinum-resistance mechanisms in ovarian cancer, since we retain the water permeability regulation of AQP1 may play an important role in drug metabolism and drugs chemo-sensitivity as elsewhere previously reported [27,28,29,30].

According to our results, we have demonstrated that high grade serous OC could be classified in two predictive groups on the basis of AQP1 expression at the time of the pre-operative diagnostic peritoneal biopsy. The first group of AQP1+ patients exhibited a poor pathological response in omental samples, indicating an eligibility for cytoreductive surgery rather than candidate for NACT. Nevertheless, the results from the present study need to be further validated on larger cohorts to establish the biological role of AQP1 as well as its clinical utility in the therapeutic approach of serous high-grade OC patients.

## Figures and Tables

**Table 1:** Patients’ Characteristics.

Case	Age	Stage	AQP1 IHC	β-Catenin IHC	CRS	Follow-Up (Months)	Outcome
1	49	IIIC	0 (Negative)	M	3	60	A
2	42	IV	0 (Negative)	M	3	60	A
3	73	IV	0 (Negative)	M	3	30	A
4	37	IIIC	0 (Negative)	MC	3	35	A
5	57	IIIC	0 (Negative)	M	3	45	A
6	55	IIIC	0 (Negative)	M	2	32	A
7	52	IIIC	0 (Negative)	M	2	24	A
8	68	IIIC	0 (Negative)	MC	2	20	D
9	45	IIIC	0 (Negative)	M	2	60	A
10	48	IV	0 (Negative)	M	2	60	A
11	71	IIIC	0 (Negative)	M	1	20	D
12	63	IIIC	0 (Negative)	M	1	30	D
13	58	IIIC	25 (Not uniform)	M	1	40	A
14	73	IIIC	25 (Not uniform)	MC	1	40	A
15	75	IIIC	25 (Not uniform)	MC	1	12	D
16	68	IIIC	25 (Not uniform)	M	1	20	D
17	46	IIIC	50 (Diffuse)	MC	1	24	A
18	49	IIIC	50 (Diffuse)	MC	1	24	A
19	55	IIIC	50 (Diffuse)	MC	1	24	A
20	61	IV	50 (Diffuse)	MC	1	32	A
21	75	IIIC	80 (Diffuse)	MC	2	24	A
22	72	IIIC	80 (Diffuse)	MC	2	20	D
23	48	IIIC	5 (Focal)	M	2	20	D
24	53	IIIC	5 (Focal)	M	2	16	D
25	57	IIIC	5 (Focal)	MC	2	26	A
26	60	IV	5 (Focal)	MC	2	40	A
27	63	IIIC	1 (Focal)	M	2	60	A
28	52	IIIC	1 (Focal)	M	2	60	A
29	59	IIIC	1 (Focal)	MC	2	30	A
30	64	IIIC	1 (Focal)	MC	2	24	D
31	66	IIIC	1 (Focal)	MC	2	34	A
32	50	IIIC	1 (Focal)	MC	2	31	A

Legend: IHC (immunohistochemistry), M (membranous), MC (membranous and cytoplasmic), CRS (complete response score), A (alive), D (dead for the disease).

**Table 2:** Distribution of CRS scores according to AQP1 staining.

CRS	AQP1–	AQP1+
1–2	7	20
3	5	0
Total patients	12	20

## Data Availability

The data presented in this study are available on request from the corresponding author.
